# Extracellular vesicles‐transmitted long non‐coding RNA MTUS2‐5 promotes proliferation and vascularization of human vascular endothelial cells in patients with Budd–Chiari syndrome

**DOI:** 10.1111/jcmm.17911

**Published:** 2023-08-18

**Authors:** Longfei Zhang, Benchi Feng, Zhuxin Zhou, Hanlin Huang, Chaowen Yu, Xiaogao Wang, Chao Xu, Yong Gao, Shiyuan Chen

**Affiliations:** ^1^ Department of Vascular Surgery The First Affiliated Hospital of Bengbu Medical College Bengbu China

**Keywords:** angiogenesis, Budd–Chiari syndrome, extracellular vesicles, HUVECs, lncRNA MTUS2‐5

## Abstract

The high rates of misdiagnosis and untreated mortality with regard to Budd–Chiari syndrome (BCS) indicated the need to screen effective biomarkers. The aim of this study was to explore the function of extracellular vesicles (EVs) in patients with BCS as well as associated mechanisms. First, differentially expressed long non‐coding RNAs (lncRNAs) from EVs separated from serum between BCS and healthy controls were screened using microarray analysis. Second, the proliferation, migration and tube formation of human vascular endothelial cells (HUVECs) were detected after EVs treatment, along with vascular endothelial growth factor (VEGF) levels and inflammatory factors from the cell supernatant. Last, the overexpressed lncRNA was transfected into the cells to further explore the mechanisms involved. Extracellular vesicles of BCS patients have significantly higher levels of lncRNA MTUS2‐5 than healthy controls. Apparently, treatment with EVs from BCS or the ones transfected with plasmids that overexpress lncRNA MTUS2‐5 enhances proliferation, migration and angiogenesis capacity. The results were considerably better than those obtained from treatment with EVs from healthy controls or transfection with the normal control plasmid, which also elevated the level of VEGF and inflammatory factors. Furthermore, FOS and PTGS2 were potentially regulated by the lncRNA MTUS2‐5 transmitted by EVs. The lncRNA MTUS2‐5 in EVs plays an important role in angiogenesis in the Budd–Chiari syndrome.

## 1 INTRODUCTION

Budd–Chiari syndrome (BCS) is mainly caused by obstruction of the hepatic vein outflow tract and inferior vena cava.^1^ Although this condition is an uncommon hepatic vascular disease, its prevalence is affected by regional disparity. Budd–Chiari syndrome has higher incidence rates in Asian than in western countries. Data presentation in China shows that there are 6.8–12 cases of BCS per 100,000 people in the middle and lower reaches of the Yellow River and Huai River.^2^ In addition, BCS was largely underdiagnosed, resulting in delayed treatment. One of the important points to note is that BCS is associated with high mortality. When untreated, 70% of BCS patients die within a year and 90% within 3 years.^3^ This suggests that early and accurate diagnosis is vital to BCS patients.

As a multifactorial disease, the aetiology and molecular mechanism of BCS are not fully understood. Thrombosis with increased blood viscosity is considered to be the most common pathophysiological mechanism.^4^ Aberrant expression of vascular endothelial growth factor (VEGF) and relative cytokines was reported in the serum of patients with membranous obstruction of the inferior vena cava (MOVC), which is the main type of BCS in China.^5^, ^6^ Therefore, the formation of the inferior vena cava diaphragm probably starts from proliferation and angiogenesis of vascular endothelial cells. However, the details of the regulated process by VEGF after endothelial injury are unknown.

Long non‐coding RNAs (lncRNAs) were defined as a class of ncRNAs with more than 200 nucleotides. It has been reported that lncRNAs play roles that are relevant in multiple cellular processes such as cell cycle progression, migration, inflammation and neovascular diseases.^7^, ^8^, ^9^ Long non‐coding RNAs can regulate inflammatory responses and signals that are involved in promoting or inhibiting adhesion molecule expression and secretion of inflammatory cytokines. Previous studies reported that lncRNA H19 participates in the process of vascular endothelial cells (VECs) injury that is induced by oxidized low‐density lipoprotein (ox‐LDL).^10^ Furthermore, lncRNA SENCR binds to cytoskeleton‐associated protein 4 to stabilize the adhesive connections between VECs. Knockout SENCR contributes to the damage of these connections, in addition to endovascular integrity, thereby promoting the permeability of VECs.^11^

Extracellular vesicles are membranous vesicles that contain various biomolecules, which include lncRNAs involved in cellular communication.^12^ It is important to note that extracellular vesicles (EVs) can be secreted by nearly all types of cells, and they exist in all body fluids such as plasma.^13^ The crosstalk between EVs and inflammasomes has been demonstrated, of which the activation of the latter regulates the release of EVs.^14^ Extracellular vesicles contain RNA and protein cargo and are a promising biomarkers in plasma.^15^ Therefore, we speculate that lncRNAs in EVs may be involved in the pathogenesis of BCS. However, isolation of high‐purity EVs is difficult since much more free proteins and lipoproteins were existed in plasma, mainly lipoproteins that have a diameter similar with EVs.^16^ Lipoproteins such as ApoA1 and ApoB participate in cholesterol and lipid transport, which play important role in angiocardiopathy. Hence, here we have to consider the role of apolipoproteins except EVs.

In the present study, we screened the targeted lncRNA in EVs and evaluated whether BCS‐EVs‐derived lncRNA could promote angiogenesis of human vascular endothelial cells. We also preliminarily explored the mechanism by which lncRNA regulates endothelial cells. We speculate that our findings may aid in providing a novel biomarker for diagnosing BCS, as well as in developing new strategies for the treatment of BCS.

## 2 MATERIALS AND METHODS

### 2.1 Patients

Budd–Chiari syndrome patients in the First Affiliated Hospital of Bengbu Medical College (China) from April 2020 to August 2021 were included in this study. The inclusion criteria included BCS patients diagnosed by CT or inferior vena cava venography and all patients who lived in northern Anhui province, China. On the other hand, patients with the following features were excluded from this study^1^: The inferior vena cava is unobstructed by iconography^2^; drug therapy was done before surgery or sample collection^3^; BCS was induced by organization oppression or trauma^4^; and presence of malignant tumours. The healthy volunteers, without genetic disease, diabetes, angiocardiopathy and tumours, were also included in the physical examination centre in our hospital. All patients and healthy controls who participated in this study were informed and signed the consent form. The Ethics Committee of Bengbu Medical College approved the study protocols.

### 2.2 Isolation and identification of extracellular vesicles

Extracellular vesicles were isolated from the serum of patients with BCS as well as from the healthy controls. First, raw serum was centrifuged at 3000 *g* for 10 min at 4°C to remove cell debris. Second, the supernatant that was obtained after centrifugation was transferred to a new centrifuge tube and centrifuged at 10,000 *g* for 10 min at 4°C to further remove impurities. Then, the supernatant was mixed with Blood PureExo Solution (BPS) on an oscillator for a minute. The mixed liquor was centrifuged at 10,000 *g* for 60 min at 4°C and, the sediments were collected. The sediments were resuspended in 1 × PBS and further centrifuged at 12,000 *g* for 2 min prior to collecting the supernatant. Last, the supernatant was transferred into the upper chamber of exosome purification filter (EPF) and centrifuged at 3000 *g* for 10 min. The purified EVs were collected from the base of EPF. To determine the purity compared to the total serum of these EVs, transmission electron microscopy (TEM) and Western blot (WB) analyses were performed. In brief, EVs were immobilized in 2.5% glutaraldehyde solution for 2 h. Next, 10 μL of the mixed solution was transferred to the copper net. Images were then acquired by TEM (JEM1230, JEOL) after dyeing with 2% phosphotungstic acid solution. For WB analyses, the total serum and isolated EVs were treated with the RIPA lysis buffer to harvest the protein supernatant. The proteins were combined and mixed with 5 × SDS before the mixture was placed in boiling water for 5 min and centrifuged at 12,000 r/min for another 5 min. The protein samples were resolved using 10% sodium dodecyl sulphate–polyacrylamide gel electrophoresis (SDS‐PAGE), after which the gel was cut according to the marker control. The first antibodies (ALIX, ab275377; TGS101, ab125011; ApoB, ab139401; ApoA1, ab151710) were incubated after PVDF transfer membrane and TBST blocking. After incubation with the secondary antibodies (Affinity; Beyotime), protein bands were visualized using the integrated chemiluminescence imager (ChemiScope 5300 Pro).

### 2.3 Microarray analysis of extracellular vesicles

Total RNA was isolated from EVs using the Trizol reagent (Invitrogen). NanoDrop ND‐2000 (Thermo Scientific) was used for quantifying the total RNA, while RNA integrity was detected by the Agilent Bioanalyzer 2100 (Agilent Technologies). The RNA was purified using the QIAGEN RNeasy Kit. The AffinityScript‐RT kit and Promoter Primer/Anti‐sense Promoter were used to inversely transcript the RNA to cDNA for library preparation. The microarray assay and analysis of the sequencing data were performed as previously described.^17^

### 2.4 Quantitative real‐time PCR (qPCR)

Total RNA was isolated using the Trizol reagent (Invitrogen), and the first strand cDNAs were synthesized using Reverse transcription kit (K1622, Thermo) according to the manufacturer's instructions. qRT‐PCR analysis was carried out using SYBRGreen PCR kit (F‐415XL, Thermo) on the 7500 Real‐Time PCR System (Applied Biosystems). The primers were designed using the Primer Premier 5.0 software and synthesized from Sangon Biotech (ShangHai). The sequences were as follows:

TMEM254‐F: 5′‐GAGTCCTTGTATGCCATAGTA‐3′,
TMEM254‐R: 5′‐ AAGCAATCAAGATGGTGAGA‐3′,
MTUS2‐5‐F: 5′‐GTGGTTCCTTCCTCCTCT‐3′,
MTUS2‐5‐R: 5′‐GCGGCTGAATCCTTAATATC‐3′.
XAGE1A‐3‐F: 5′‐GAGCCTCAACAAGAAGAAC‐3′.
XAGE1A‐3‐R: 5′‐ GCCAACTCCACATTCATC‐3′,
FAM135B‐4‐F: 5′‐ ATGAGGTGGACAGAAGGTT‐3′,
FAM135B‐4‐R: 5′‐ CTCTCCTGCTTTGCCCTA‐3′,
GAPDH‐F: 5′‐GGAGCGAGATCCCTCCAAAAT‐3′,
GAPDH‐R: 5′‐GGCTGTTGTCATACTTCTCATGG‐3′.

The fold change on expression was calculated by the 2^˗△△ct^ method.

### 2.5 Cell culture and treatment

The human umbilical vein endothelial cell (HUVEC) was purchased by the China Type Culture Collection (CTCC) and cultured in Roswell Park Memorial Institute 1640 medium (Hyclone) with 10% foetal bovine serum (Lonsera) and 1% penicillin/streptomycin. Cells were incubated in the thermostatic incubator under conditions of 5% CO_2_ and 37°C. Human umbilical vein endothelial cell cells were co‐cultured with EVs (100 μg/mL) from BCS patients and the healthy control for 48 h, respectively.

### 2.6 Extracellular vesicles labelling and uptake

To track the uptake of EVs by HUVECs, the former were labelled using PKH26 red fluorescent dye (UR52302, Umibio), while Hoechst (C1022, Beyotime) was used to label nuclei. The HUVECs were cultured in 48‐well plates at 1 × 10^5^ cells/mL for 24 h. The PKH‐26 labelling EVs were added into the HUVECs at a concentration of 100 μg/mL. Human umbilical vein endothelial cells were then placed into the incubator, and a fluorescence microscope (IX73, OLYMPUS) was used to capture images at 2‐, 4‐, 8‐ and 24‐h observation points, respectively.

### 2.7 Cell transfection

For mechanism studies, the HUVEC cells were transfected with overexpressing lncRNA MTUS2‐5 and the empty vector control (Sangon Biotech) using Lipofectamine 2000 (Beyotime Biotechnology), following the manufacturer's instructions.

### 2.8 RNA sequencing

Total RNA was isolated from the HUVECs, and it was reverse transcribed into cDNA to establish the PCR library. The size and concentration of the library were detected using the Agilent 2100 Bioanalyzer and fluorescent quantitation. RNA sequencing was performed by the Illumina HiSeq platform according to the instructions from the manufacturer.

### 2.9 Enzyme‐linked immunosorbent assay

The supernatant from the HUVECs was centrifuged (3000 rpm/min, 20 min), and the cell‐free supernatant was collected into centrifuge tubes. The enzyme‐linked immunosorbent assay (ELISA) technique (Ybio) was used to detect the levels of the vascular inflammatory factor. The standard curve of each factor was applied to calculate the concentrations of the analytes. The microplate reader (MK3, Thermo) was employed in measuring absorbance at 450 nm.

### 2.10 Western blotting

The total RNA was isolated from HUVECs using the RIPA lysate, and quantification was done using the BCA kit (Biosharp). The primary antibody p65/p‐p65 (ab32536/ab76302, abcam) with the housekeeping antibody mouse‐derived GAPDH (ab8245, abcam) was used to detect the levels of inflammatory proteins. The procedure was performed according to the afore‐mentioned methods.

### 2.11 Detection of cell proliferation

Cell proliferation was assessed using a Cell Counting Kit‐8 (CCK8, Beyotime Biotechnology) kit, based on the manufacturer's instructions. In brief, cells were seeded in 96‐well plates at a density of 2 × 10^5^ cells per well before they were cultured in an incubator for 24 h. After treatment with EVs or lncRNA MTUS2‐5 transfection for 48 h, 20 μL CCK8 was added, and cells were further cultured for 3 h. The optical density (OD) at 450 nm was measured using the microplate reader (MK3, Thermo).

### 2.12 Cell migration assay

The scratch test was used to assess cell migration. Human vascular endothelial cells were seeded in six‐well plates at a density of 2 × 10^5^ cells per well and were cultured overnight. After treatment with EVs or plasmid transfection for 48 h, 5 × 10^5^ cells were obtained and cultured in a 3.5‐cm dish overnight. This followed after cell dissociation using 0.25% trypsin. A scratch was made using a sterile 200‐μL pipette tip in the centre of the cell dishes when the density reached 90%. After washing with PBS three times, cells were cultured for 24 h. We observed the photographs at 0 and 24 h using the OLYMPUS microscope (IX71). The ImageJ software was used to quantify the migration distance.

### 2.13 Tube forming test

Pre‐cooled nine matrigel was added to the 24‐well plates at a volume of 250 μL followed by further incubation at 37°C for 30 min to form a soil gel. Human vascular endothelial cells were seeded in the 24‐well plates with matrigel at a density of 5 × 10^4^ cells per well, and further culturing was done in complete medium (RPMI 1640 containing 10% FBS and 1% penicillin–streptomycin) for 8 h. Tubule formation was observed using the OLYMPUS (IX71) microscope. Further analysis was done by measuring branch length and counting tubule numbers using the ImageJ software.

### 2.14 Statistical analysis

SPSS 26.0 version and GraphPad Prism 8 were used to analysis the data. All data were presented as mean ± standard deviation (SD) and showing as dot plots. Student's *t*‐test was performed to compare the statistical difference between two groups, while one‐way analysis of variance (anova) was used for compassion between multiple groups. *p* < 0.05 was considered as a statistically significant difference.

## 3 RESULTS

### 3.1 Isolation and identification of serum extracellular vesicles

**FIGURE 1:**
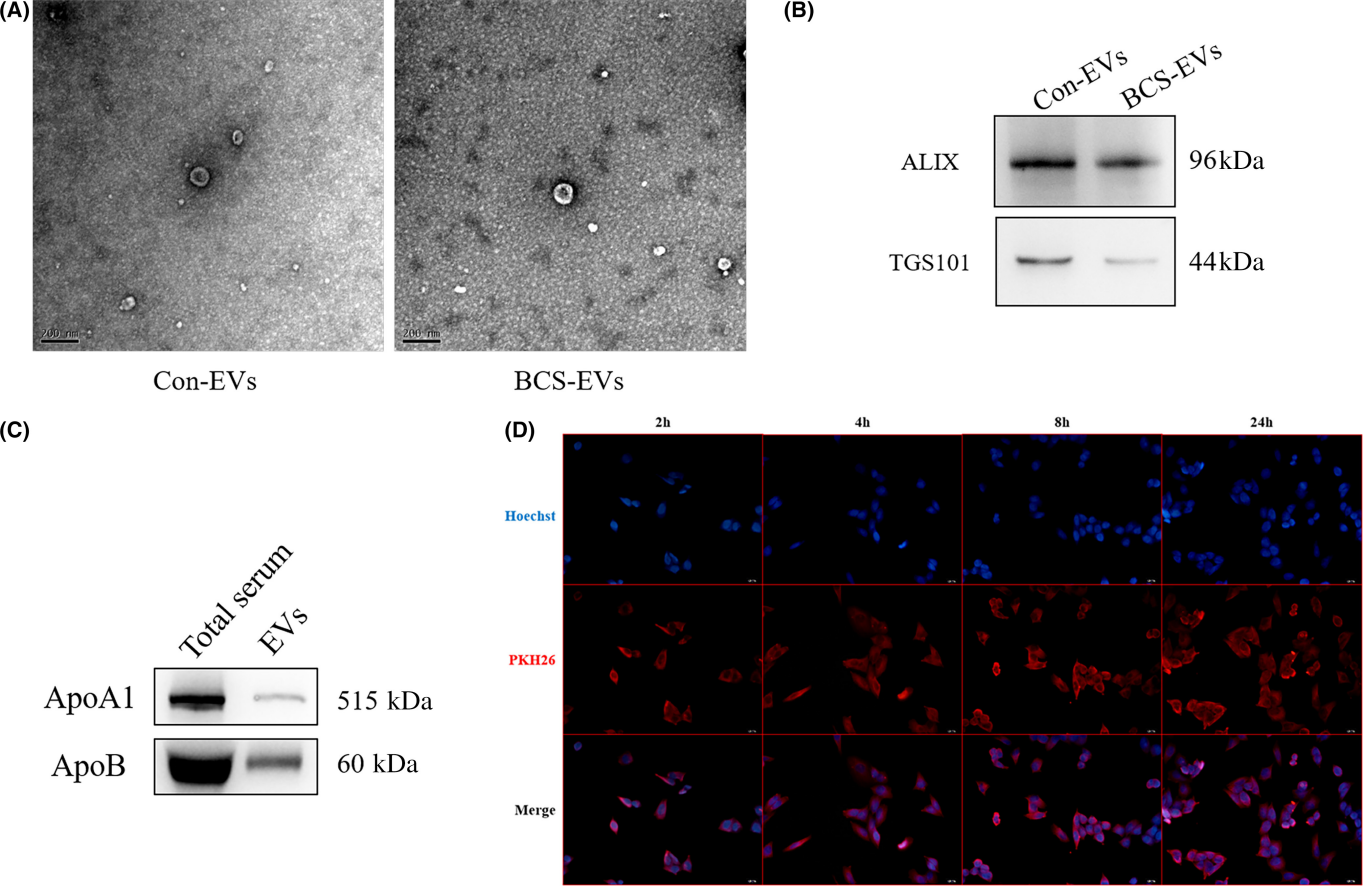
The identification and labelling of extracellular vesicles (EVs). (A) The morphology of EVs observed by the transmission electron microscope; (B) the marked proteins of EVs detected by Western blot analysis; (C) the ApoA1 and ApoB were detected by Western blot analysis; (D) the labelling EVs and those uptaken by human vascular endothelial cells were detected by immunofluorescence.

Extracellular vesicles were isolated from the serum of BCS patients (BCS‐EVs) and healthy volunteers (Con‐EVs). The morphology of EVs was observed using the TEM where EVs exhibited cystic spherical shape (Figure 1A). The marked proteins of EVs (ALIX and TGS101) were identified by WB, and the results showed that all the two markers were detected in EVs (Figure 1B). These results suggested that the isolated microvesicles from the serum of BCS patients and healthy controls were EVs. Furthermore, protein expressions of ApoB and ApoA1 were detected in EVs and the total serum to determine the purity of the EVs. The results revealed that both the total serum and EVs had expressions of the proteins, although the level in EVs was much lower than that in the total serum (Figure 1C).

### 3.2 The effect of BCS‐EVs on human vascular endothelial cells

**FIGURE 2:**
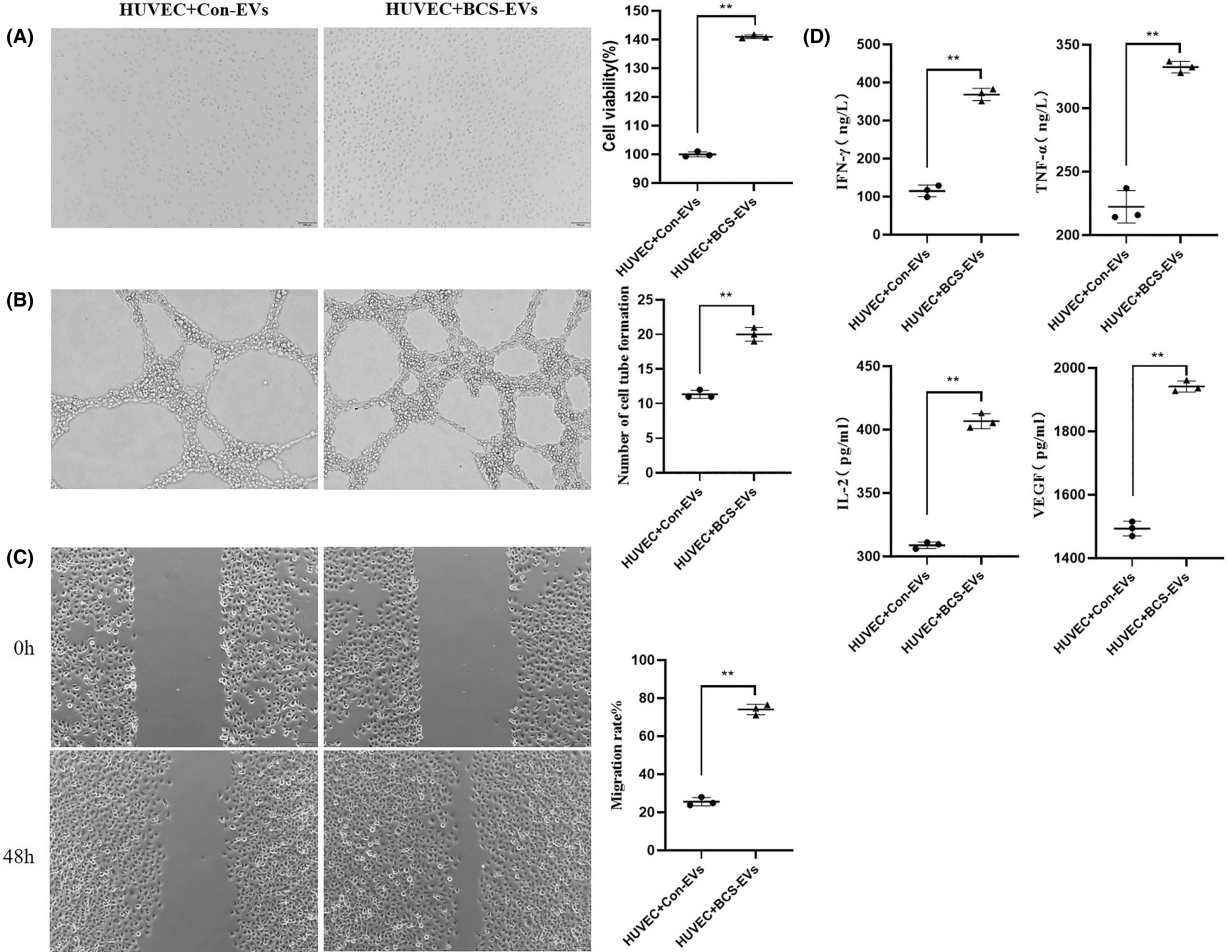
The regulated function of extracellular vesicles (EVs) that were derived from Budd–Chiari syndrome (BCS) patients. The regulated function of proliferation (A), angiopoiesis (B), migration (C) and inflammatory cytokines levels (D) by BCS‐EVs on human vascular endothelial cells. **p < 0.01 indicated a significant difference between the two groups.

To determine the effects of BSC‐EVs on HUVECs, the fusion between the PKH26 labelling EVs and HUVECs was initially observed. As shown in Figure 1D, HUVECs gradually engulfed the EVs in a time‐dependent manner. In addition, many EVs entered HUVECs and accumulated around the nucleus after 24 h of co‐cultivation. Moreover, CCK8, Transwell assays, and tube formation were performed in HUVECs. The results showed that BCS‐EVs significantly increased the proliferative capability of HUVECs, compared with Con‐EVs (Figure 2A). Meanwhile, BSC‐EVs increased the number of vascular branches in HUVECs relative to Con‐EVs, and this indicated a pro‐angiogenetic role of BSC‐EVs (Figure 2B). Moreover, the migration ability of HUVECs was significantly improved after treatment with BSC‐EVs compared to Con‐EVs treatment (Figure 2C). In addition, the VEGF and inflammatory factors from the supernatant of HUVECs were detected using the ELISA kit. Observations showed that the levels of VEGF, TNF‐a, IL‐2 and IFN‐γ were significantly higher when treatments were done with BCS‐EVs compared to Con‐EVs (Figure 2D). All suggest that EVs from the serum of patients with BCS could promote the angiogenesis of HUVECs.

### 3.3 The regulated mechanism of lnc MTUS2‐5 on human vascular endothelial cells

**FIGURE 3:**
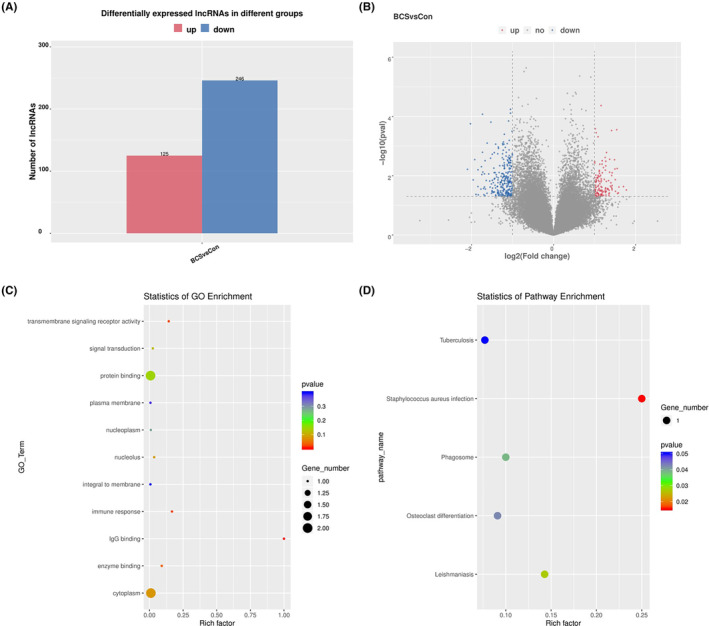
Microarray analysis showing the differentially expressed lncRNA between extracellular vesicles from the healthy control and Budd–Chiari syndrome patients. The differentially expressed lncRNAs were shown by a histogram (A) and volcano plot (B). The GO (C) and KEGG (D) enrichment analyses were presented as scatter diagrams.

**FIGURE 4:**
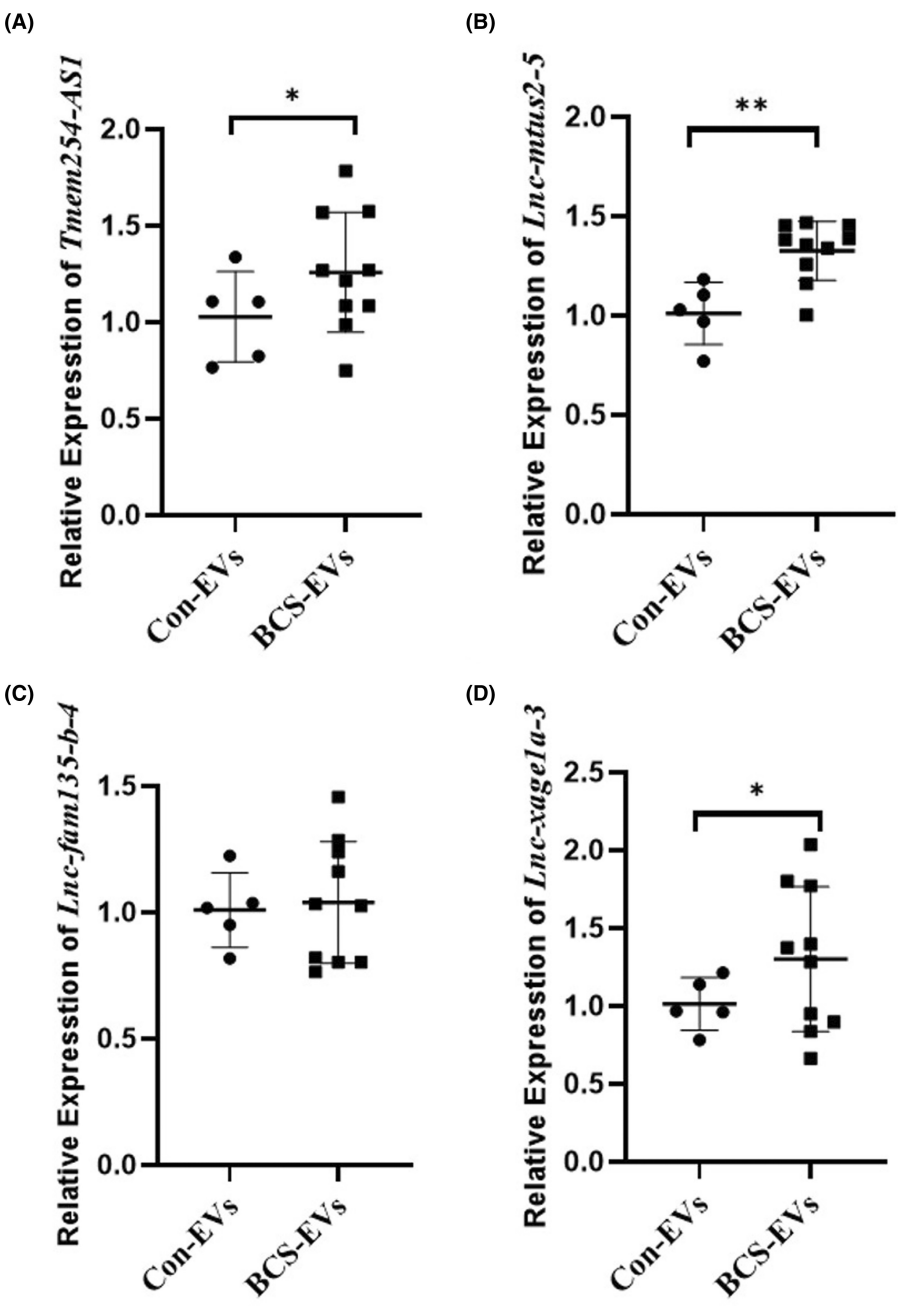
Four long non‐coding RNAs were detected between extracellular vesicles from healthy controls and Budd–Chiari syndrome patients by qPCR. *p < 0.05 and **p < 0.01 indicated a significant difference between the two groups.

To investigate the potential lncRNA that may regulate the role of EVs on HUVEC angiogenesis, a microarray analysis was performed to detect the lncRNAs profile of EVs between BCS patients and healthy controls. A total of 371 differentially expressed lncRNAs were obtained, with 125 upregulated and 246 downregulated ones (Figure 3A,B). Moreover, the GO and KEGG enrichment analyses were performed to explore the function and pathways of the differentially expressed lncRNAs (Figure 3C,D). Four were selected from these highly expressed lncRNAs for further analysis, according to the *p* value, and these were lnc‐XAGE1A, MTUS2‐5, TMEM254 and FAM135B‐4. The results from qPCR showed that lncRNA XAGE1A, MTUS2‐5 and TMEM254 were highly expressed to a significant extent in EVs from the serum of BCS patients compared to the healthy controls, with MTUS2‐5 being the most frequently expressed. No statistical significance was noted in lncRNA FAM135B‐4 (Figure 4A–D). Hence, lncRNA MTUS2‐5 was included as the targeted molecule that might play an important role in the regulation of BCS.

**FIGURE 5:**
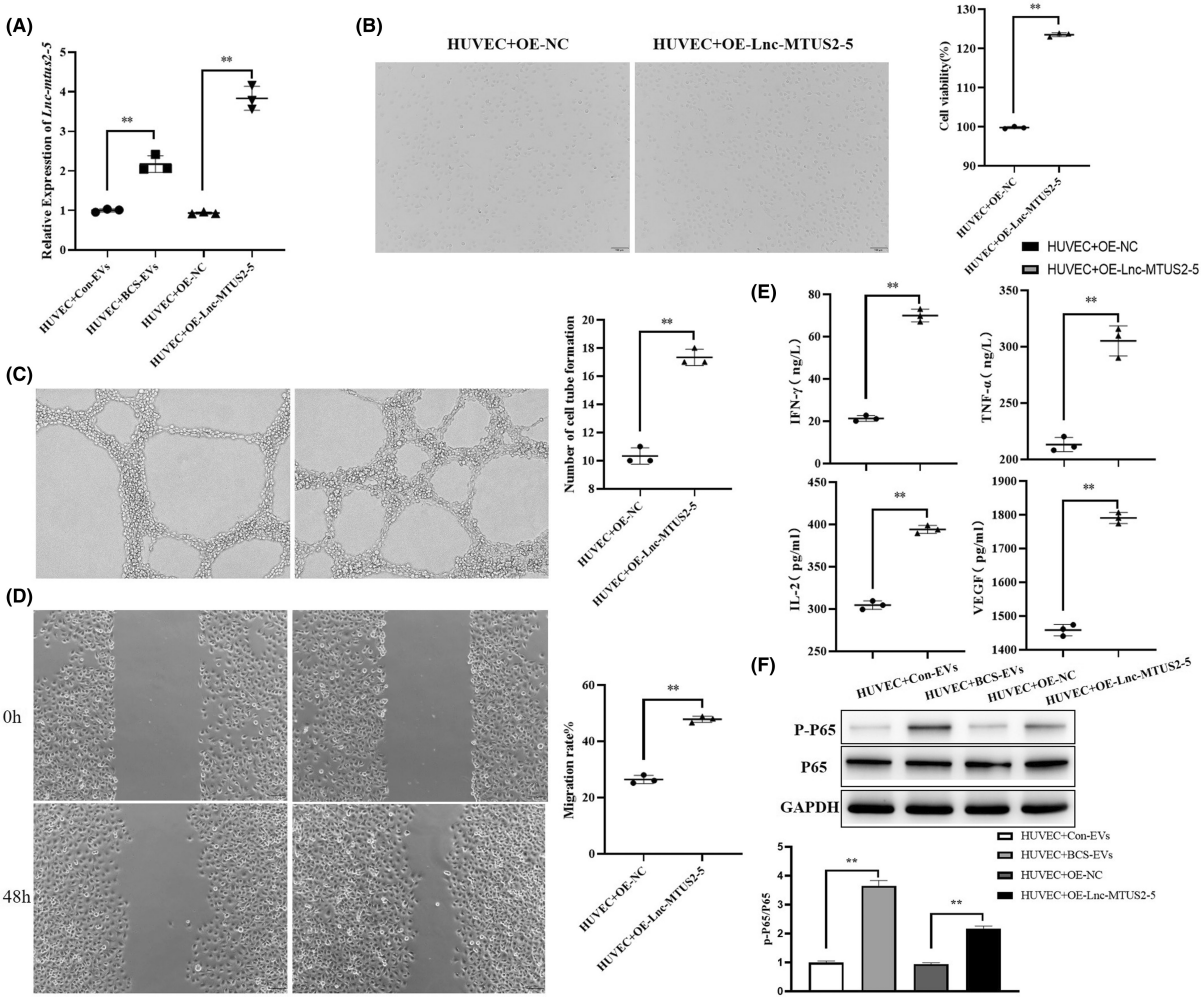
The regulated function of overexpressing lnc‐MTUS2‐5 on human vascular endothelial cells (HUVECs). The verification of treatment by BCS‐EVs and overexpressing lnc‐MTUS2‐5 in HUVECs (A). The regulated function of proliferation (B), angiopoiesis (C), migration (D) and inflammatory cytokines (E) by transfected normal and overexpressing lnc‐MTUS2 on HUVECs. The change of p‐P65/P65 in HUVECs was detected using the Western blot analysis after treatment with extracellular vesicles (EVs) from healthy controls and Budd–Chiari syndrome (BCS) patients or transfecting with normal and overexpressing lnc‐MTUS2 (F). **p < 0.01 indicated a significant difference between the two groups.

To further determine whether the lncRNA MTUS2‐5 from BSC‐EVs had a positive role on HUVECs, its level was detected after treatment with BSC‐EVs. The results showed that lncRNA MTUS2‐5 was highly expressed in HUVECs that were treated with BCS‐EVs than with Con‐EVs (Figure 5A). The overexpressing lncRNA MTUS2‐5 or NC plasmid was then transfected into HUVECs. The qPCR assay verified the high expression of MTUS2‐5 in the OE‐group compared to NC‐group (Figure 5A). Phenotype assays revealed that overexpression of MTUS2‐5 significantly promoted cell proliferation and tube formation in HUVECs, in addition to enhancing cell migration (Figure 5B–D). On the other hand, the results from the ELISA assay showed that the levels of VEGF, TNF‐a, IL‐2 and IFN‐γ were significantly higher in the supernatant of HUVECs with OE‐MUST2‐5 transfection compared to NC‐transfected cells (Figure 5E). To further study the inflammatory reaction induced by lncRNA MTUS2‐5 on HUVECs, a change of the NF‐kB pathway was detected after the cells were treated with BCS‐EVs or OE‐MUST2‐5. We found that p‐P65 was highly expressed to a significant extent in HUVECs that were treated with BSC‐EVs or OE‐MUST2‐5 transfection, than in cells that were subjected to Con‐EVs or OE‐NC transfection (Figure 5F). This suggests that NF‐kB signalling can be activated by lncRNA MTUS2‐5 in HUVECs.

**FIGURE 6:**
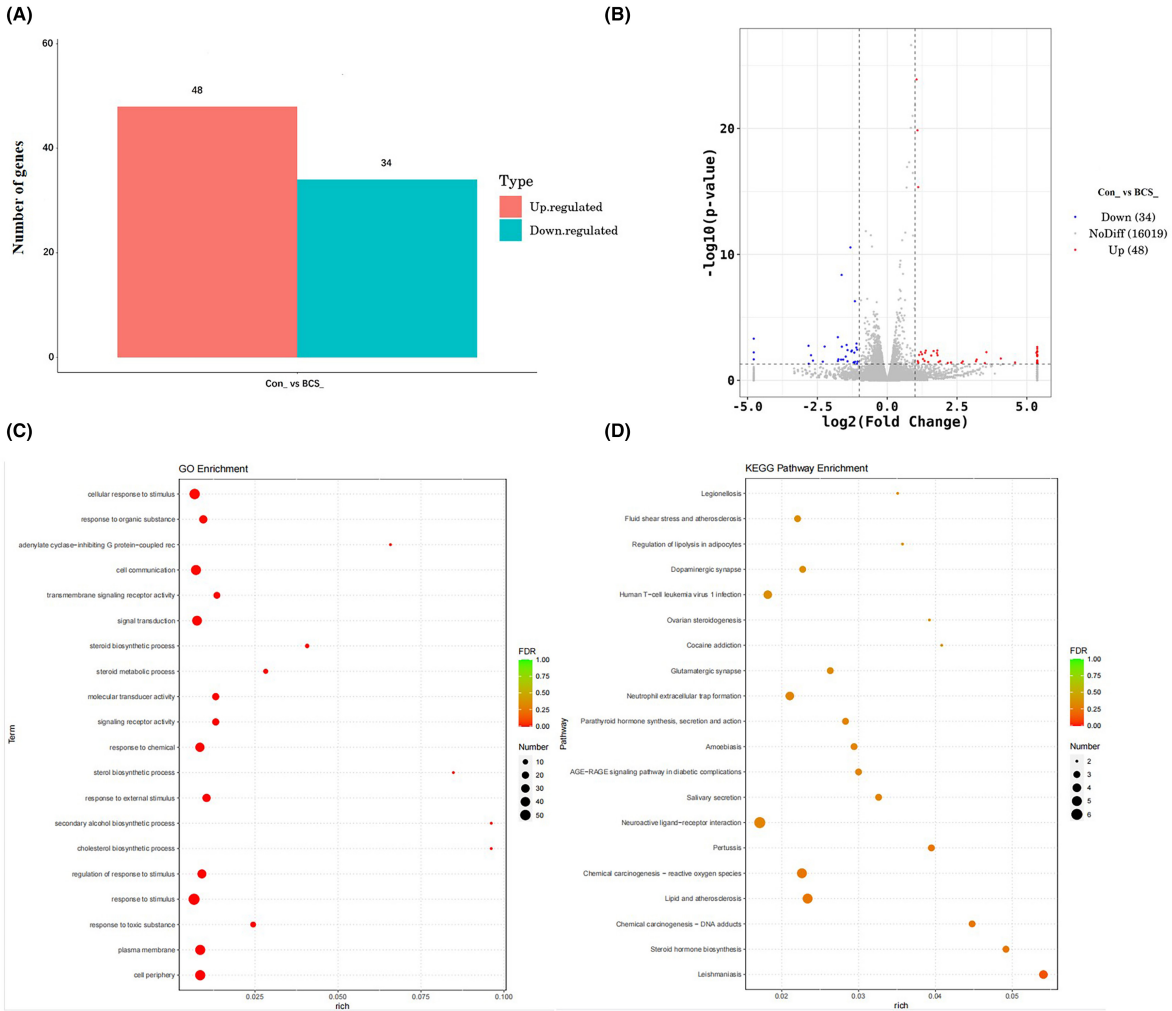
The potential genes and signals in human vascular endothelial cells (HUVECs) regulated by BCS‐EVs were analysed using RNA‐sequencing technology. The differentially expressed mRNAs between HUVECs treated by extracellular vesicles (EVs) derived from healthy controls, and Budd–Chiari syndrome (BCS) patients were shown by histogram (A) and volcano plot (B). The GO (C) and KEGG (D) enrichment analyses were presented in the form of scatter diagrams.

**FIGURE 7:**
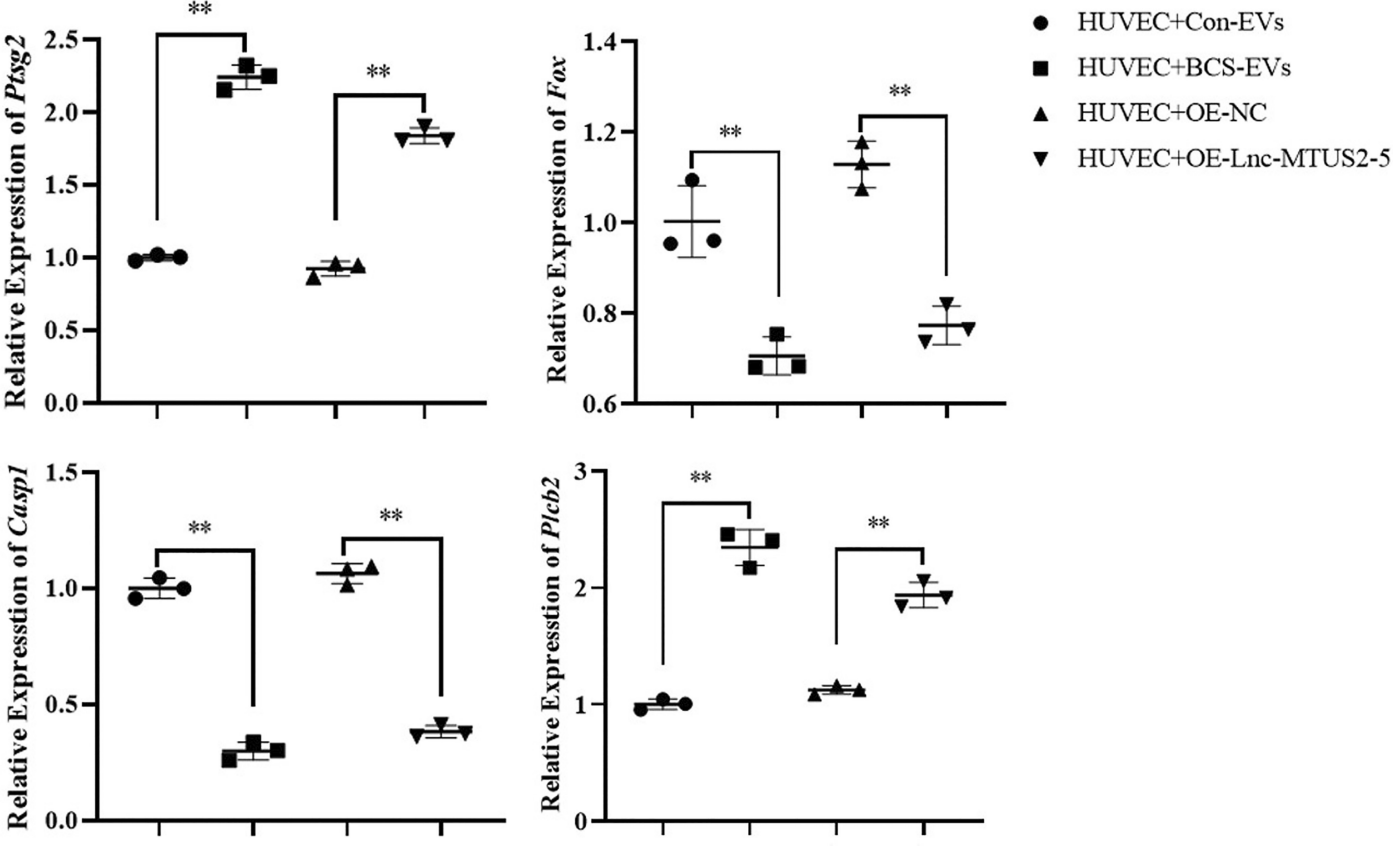
The targeted genes regulated by extracellular vesicles derived from Budd–Chiari syndrome patients or overexpressing lnc‐MTUS2‐5 in human vascular endothelial cells were verified by qPCR. **p < 0.01 indicated a significant difference between the two groups.

To further explore the regulated mechanism of BCS‐EVs on HUVECs, RNA sequencing was performed to predict the potential target genes and signals. Based on the results revealed, a total of 82 differentially expressed genes (DEGs) were obtained (48 upregulated and 34 downregulated genes) between BCS‐EVs and Con‐EVs‐treated HUVECs (Figure 6A,B), with the criterion of *p* < 0.05 and |log2FoldChange| > 1. Moreover, GO and KEGG enrichment analyses were performed to analyse the relative functions and pathways of these differentially expressed genes. The results suggested that the DEGs were mainly enriched in cellular response to stimulus function, fluid shear stress and atherosclerosis and lipolysis adipocytes pathways (Figure 6C,D). We further demonstrated gene expression in these pathways. When compared to the HUVECs that were treated with Con‐EVs or the OE‐NC‐transfected cells, FOS and PTGS2 genes showed significantly lower and higher expression in BCS‐EVs or OE‐MTUS2‐5 transfected HUVECs, respectively (Figure 7). Overall, the results from this study revealed several molecular mechanisms that are regulated by EVs or lncRNA MTUS2‐5 on the HUVECs in patients with BCS.

## 4 DISCUSSION

In the present study, we first demonstrated that the EVs separated from patients with BCS could promote the proliferation, migration and tube formation of HUVECs in vitro. We also highlighted that the EVs increase the levels of VEGF and inflammatory factors in the supernatant of HUVECs. This indicated that the BCS‐EVs have an important role in the regulation of HUVECs. Furthermore, we obtained the differentially expressed lncRNAs profile in EVs from the serum of patients with BCS compared to the healthy controls using the microarray technology. Moreover, lncRNA MTUS2‐5 was screened as a targeted molecule that promotes the proliferation, migration and tube formation of HUVECs. In addition, overexpressing lncRNA MTUS2‐5 also increased the level of VEGF and inflammatory factors in the supernatant of HUVECs, a result that suggests that lncRNA MTUS2‐5 has an important function in regulating HUVECs. Using RNA‐sequencing technology, we further discovered the differentially expressed genes that are regulated by BCS‐EVs. Combined enrichment analysis, FOS and PTGS2 were selected as the targeted genes of MTUS2‐5 in HUVEC. This also indicated that atherosclerosis and lipolysis adipocytes pathways were involved in the regulation of HUVECs.

Budd–Chiari syndrome is a complex condition that involves multiple factors, and its pathogenesis and epidemiology exhibit marked differences between distinct regions.^18^ In Western countries, thrombogenesis is the most common aetiology. However, MOVC is the most prevalent among all types of BCS in China.^19^ Reports show that the surface of the inferior vena cava septum covered with endothelial cells, and vascular endothelial tissues are the main ingredients on the surface of the membrane.^20^ Therefore, the abnormal proliferation and angiogenesis of HUVECs are the key factors involved in membranous formation. Our study demonstrated that EVs from the serum of BCS patients could transmit lncRNA MTUS2‐5 to regulate the proliferation and angiogenesis of HUVECs, thereby causing the obstruction of the inferior vena cava. lncRNA MTUS2‐5 in EVs may be a promising diagnostic marker in BCS.

To the best of our knowledge, there are no reports of the involvement of EVs in BCS in previous studies. Extracellular vesicles participate in multiple physiological and pathological processes, such as tissue repair and regulation of infection.^21^ It has been reported that EVs can initiate and promote angiogenesis by targeting endothelial cells or by interaction with immune cells.^22^ Extracellular vesicles that are derived from tumour cells or serum of tumour patients can be absorbed by normal endothelial cells, thereby resulting in angiogenesis.^23^ On the other hand, Hui Xu's^24^ study found that EVs that derived miRNA from epithelial cells were involved in regulating the epithelium and fibroblast. These studies indicated that EVs play an important role in the regulation of angiogenesis in several pathological processes. However, the function of these EVs on angiogenesis might partly be attributed to the presence of apolipoproteins since lipoproteins participate in several known pro‐angiogenic effects.^25^ Our study showed that EVs along with apolipoproteins from the serum of patients with BCS lead to the angiogenesis of HUVECs, thereby providing a new mechanism and diagnostic marker for BCS. However, the source of the EVs in the regulation of angiogenesis in BCS was unknown. Ribeiro‐Rodrigues, T. M.'s^26^ reports found that EVs that are secreted by myocardial cells under ischemia induce angiogenesis. We speculate that the EVs with high levels of lncRNA MTUS2‐5 were possibly secreted by the damaged endothelial cells or smooth muscle cells and further absorbed by HUVECs to promote angiogenesis in the vena cava.

Although lncRNAs were involved in multiple pathological process, which include the physiological function and injury of vascular endothelial cells through lncRNA‐protein interaction or ceRNA (competitive endogenous RNA) mechanism.^11^, ^27^, ^28^, ^29^, ^30^ However, if and how lncRNAs regulate the endothelial cells under BCS were not clear. We demonstrated a series of lncRNAs that were abnormally expressed in EVs from patients with BCS. We also showed the regulated function of lncRNA MTUS2‐5 on HUVECs in vitro. Previous reports demonstrated that elevated VEGF levels existed in the blood of patients whose inferior vena cava is obstructed.^31^ Vascular endothelial growth factor can modulate the proliferation and angiogenesis of vascular endothelial cells and significantly correlate with thrombogenesis.^32^ A study by Mengdie Xu's^5^ team found that VEGF is a target for the regulation of HUVECs. The results from our study present the high secretion of VEGF from HUVECs when treated with overexpressing lncRNA MTUS2‐5 transfection or EVs from BCS patients, which is consistent with these previous studies. The findings also suggested that VEGF is a potential molecular target of lncRNA MTUS2‐5 in the process of angiogenesis under BCS.

Some limitations existed during the course of this study. First, we did not verify that the EVs carrying lncRNA MTUS2‐5 were derived from the damaged vascular endothelial cells in BCS. Second, the details of the mechanisms that MTUS2‐5‐regulated angiogenesis of HUVECs need to be further studied. Overall, this study provides a potential EVs diagnostic marker and its preliminary mechanism in the regulation of BCS.

## AUTHOR CONTRIBUTIONS

**longfei Zhang:** Data curation (equal); methodology (equal); software (equal); writing – original draft (lead); writing – review and editing (equal). **Benchi Feng:** Data curation (equal); formal analysis (equal); investigation (equal); methodology (equal); writing – original draft (supporting). **Zhuxin Zhou:** Data curation (equal); formal analysis (equal); methodology (supporting); resources (equal); validation (equal). **Hanlin Huang:** Investigation (supporting); project administration (equal); software (equal); validation (equal); visualization (equal). **Chaowen Yu:** Data curation (equal); supervision (equal). **Xiaogao Wang:** Resources (equal); software (equal). **Chao Xu:** Software (equal); validation (equal); visualization (equal). **Yong Gao:** Supervision (equal). **Shiyuan Chen:** Conceptualization (lead); funding acquisition (lead); supervision (equal).

## FUNDING INFORMATION

This study was supported by the Key Project of Natural Science research in Universities of Anhui Province (Grant number: KJ2020A0558); Natural Science Key Project of Bengbu Medical College (Grant number: 2021byzd168); Major Natural Science Project of Colleges and Universities in Anhui Province (KJ2016SD38) and Anhui Science and Technology Project (Grant number: 1704a0802160).

## CONFLICT OF INTEREST STATEMENT

The authors declare that they have no competing interests, and all authors confirm accuracy.

## Data Availability

The data used to support the findings of this study are available and can be provided by the corresponding author upon request.

## References

[1] Zanetto A , Pellone M , Senzolo M . Milestones in the discovery of Budd–Chiari syndrome. Liver Int. 2019;39(7):1180‐1185. 30843330 10.1111/liv.14088

[2] Zhang W , Qi X , Zhang X , et al. Budd–Chiari syndrome in China: a systematic analysis of epidemiological features based on the Chinese literature survey. Gastroenterol Res Pract. 2015;2015:738548. 26504461 10.1155/2015/738548 PMC4609452

[3] Fu Y , Sun YL , Ma XX , et al. Necessity and indications of invasive treatment for Budd–Chiari syndrome. Hepatobiliary Pancreat Dis Int. 2011;10(3):254‐260. 21669567 10.1016/s1499-3872(11)60042-8

[4] Valla D , Casadevall N , Lacombe C , et al. Primary myeloproliferative disorder and hepatic vein thrombosis. A prospective study of erythroid colony formation in vitro in 20 patients with Budd–Chiari syndrome. Ann Intern Med. 1985;103(3):329‐334. 4026081 10.7326/0003-4819-103-3-329

[5] Xu M , Cao L , Zhang X , et al. miR‐3133 inhibits proliferation and angiogenesis by targeting the JUNB/VEGF pathway in human umbilical vein endothelial cells. Oncol Rep. 2020;44(4):1699‐1708. 32945523 10.3892/or.2020.7715

[6] Dang X , Li L , Xu P . Research status of Budd–Chiari syndrome in China. Int J Clin Exp Med. 2014;7(12):4646‐4652. 25663961 PMC4307408

[7] Li J , Tian H , Yang J , Gong Z . Long noncoding RNAs regulate cell growth, proliferation, and apoptosis. DNA Cell Biol. 2016;35(9):459‐470. 27213978 10.1089/dna.2015.3187

[8] Bhan A , Soleimani M , Mandal SS . Long noncoding RNA and cancer: a new paradigm. Cancer Res. 2017;77(15):3965‐3981. 28701486 10.1158/0008-5472.CAN-16-2634 PMC8330958

[9] Conte I , Banfi S , Bovolenta P . Non‐coding RNAs in the development of sensory organs and related diseases. Cell Mol Life Sci. 2013;70(21):4141‐4155. 23588489 10.1007/s00018-013-1335-z PMC11113508

[10] Cao L , Zhang Z , Li Y , Zhao P , Chen Y . LncRNA H19/miR‐let‐7 axis participates in the regulation of ox‐LDL‐induced endothelial cell injury via targeting periostin. Int Immunopharmacol. 2019;72:496‐503. 31054453 10.1016/j.intimp.2019.04.042

[11] Ye F , Zhang J , Zhang Q , Zhang J , Chen C . Preliminary study on the mechanism of long noncoding RNA SENCR regulating the proliferation and migration of vascular smooth muscle cells. J Cell Physiol. 2020;235(12):9635‐9643. 32401347 10.1002/jcp.29775

[12] Liu J , Ren L , Li S , et al. The biology, function, and applications of exosomes in cancer. Acta Pharm Sin B. 2021;11(9):2783‐2797. 34589397 10.1016/j.apsb.2021.01.001 PMC8463268

[13] Tang XH , Guo T , Gao XY , et al. Exosome‐derived noncoding RNAs in gastric cancer: functions and clinical applications. Mol Cancer. 2021;20(1):99. 34330299 10.1186/s12943-021-01396-6 PMC8323226

[14] Noonin C , Thongboonkerd V . Exosome‐inflammasome crosstalk and their roles in inflammatory responses. Theranostics. 2021;11(9):4436‐4451. 33754070 10.7150/thno.54004 PMC7977448

[15] Shah R , Patel T , Freedman JE . Circulating extracellular vesicles in human disease. N Engl J Med. 2018;379(10):958‐966. 30184457 10.1056/NEJMra1704286

[16] Simonsen JB . What are we looking at? Extracellular vesicles, lipoproteins, or both? Circ Res. 2017;121(8):920‐922. 28963190 10.1161/CIRCRESAHA.117.311767

[17] Huang X , Sun L , Wen S , et al. RNA sequencing of plasma exosomes revealed novel functional long noncoding RNAs in hepatocellular carcinoma. Cancer Sci. 2020;111(9):3338‐3349. 32506598 10.1111/cas.14516 PMC7469810

[18] Qi X , Guo X , Fan D . Difference in Budd–Chiari syndrome between the West and China. Hepatology. 2015;62(2):656. 25476543 10.1002/hep.27628

[19] Zu M , Xu H , Zhang Q , et al. Review of Budd–Chiari syndrome. J Interv Med. 2020;3(2):65‐76. 34805910 10.1016/j.jimed.2020.03.002 PMC8562171

[20] Teng F , Zu MH , Hua QJ . Correlations of iodide ions with vascular endothelial growth factor and its receptors during the proliferation of vascular endothelial cells. Genet Mol Res. 2014;13(3):6439‐6447. 25158262 10.4238/2014.August.25.7

[21] Olejarz W , Kubiak‐Tomaszewska G , Chrzanowska A , Lorenc T . Exosomes in angiogenesis and anti‐angiogenic therapy in cancers. Int J Mol Sci. 2020;21(16):5840. 32823989 10.3390/ijms21165840 PMC7461570

[22] Whiteside TL . Tumor‐derived exosomes and their role in cancer progression. Adv Clin Chem. 2016;74:103‐141. 27117662 10.1016/bs.acc.2015.12.005 PMC5382933

[23] Ludwig N , Whiteside TL . Potential roles of tumor‐derived exosomes in angiogenesis. Expert Opin Ther Targets. 2018;22(5):409‐417. 29634426 10.1080/14728222.2018.1464141 PMC6126896

[24] Xu H , Ling M , Xue J , et al. Exosomal microRNA‐21 derived from bronchial epithelial cells is involved in aberrant epithelium‐fibroblast cross‐talk in COPD induced by cigarette smoking. Theranostics. 2018;8(19):5419‐5433. 30555555 10.7150/thno.27876 PMC6276085

[25] Primer KR , Psaltis PJ , Tan JTM , Bursill CA . The role of high‐density lipoproteins in endothelial cell metabolism and diabetes‐impaired angiogenesis. Int J Mol Sci. 2020;21(10):3633. 32455604 10.3390/ijms21103633 PMC7279383

[26] Ribeiro‐Rodrigues TM , Laundos TL , Pereira‐Carvalho R , et al. Exosomes secreted by cardiomyocytes subjected to ischaemia promote cardiac angiogenesis. Cardiovasc Res. 2017;113(11):1338‐1350. 28859292 10.1093/cvr/cvx118

[27] Bridges MC , Daulagala AC , Kourtidis A . LNCcation: lncRNA localization and function. J Cell Biol. 2021;220(2):e202009045. 33464299 10.1083/jcb.202009045 PMC7816648

[28] Bian W , Jing X , Yang Z , et al. Downregulation of LncRNA NORAD promotes ox‐LDL‐induced vascular endothelial cell injury and atherosclerosis. Aging. 2020;12(7):6385‐6400. 32267831 10.18632/aging.103034 PMC7185106

[29] Ferrè F , Colantoni A , Helmer‐Citterich M . Revealing protein‐lncRNA interaction. Brief Bioinform. 2016;17(1):106‐116. 26041786 10.1093/bib/bbv031 PMC4719072

[30] Huang Z , Winata WA , Zhang K , et al. Reconstruction of a lncRNA‐associated ceRNA network in endothelial cells under circumferential stress. Cardiol Res Pract. 2020;2020:1481937. 32148949 10.1155/2020/1481937 PMC7042510

[31] Han XQ , Mao‐Heng ZU . Study the significance of VEGF abnormal expression in membranous obstruction with Budd–Chiari syndrome. Contemporary Medicine. 2010;16:672‐674.

[32] Chamorro‐Jorganes A , Lee MY , Araldi E , et al. VEGF‐induced expression of miR‐17‐92 cluster in endothelial cells is mediated by ERK/ELK1 activation and regulates angiogenesis. Circ Res. 2016;118(1):38‐47. 26472816 10.1161/CIRCRESAHA.115.307408 PMC4703066

